# Toxicity Assessment of Mixed Exposure of Nine Perfluoroalkyl Substances at Concentrations Relevant to Daily Intake

**DOI:** 10.3390/toxics12010052

**Published:** 2024-01-10

**Authors:** Kazuki Takeda, Taki Saito, Sakura Sasaki, Akifumi Eguchi, Makoto Sugiyama, Saeka Eto, Kio Suzuki, Ryo Kamata

**Affiliations:** 1 Laboratory of Toxicology, School of Veterinary Medicine, Kitasato University, E23-35-1, Towada, Aomori 034-0021, Japan; 2 Department of Computer Science, Tokyo Institute of Technology, 4259-J3-1818, Nagatsuta-cho, Midori-ku, Yokohama-shi 226-0026, Kanagawa, Japan; 3 Laboratory of Toxicology, Department of Environmental Veterinary Sciences, Faculty of Veterinary Medicine, Hokkaido University, N18 W9, Kita-ku, Sapporo 060-0818, Japan; 4 Department of Sustainable Health Science, Center for Preventive Medical Sciences, Chiba University, Chiba 263-8522, Japan; 5 Laboratory of Anatomy, School of Veterinary Medicine, Kitasato University, Aomori 034-0021, Japan

**Keywords:** poly-fluoroalkyl substances, liver toxicity, multi-omics analysis, environmentally relevant exposure

## Abstract

Per- and poly-fluoroalkyl substances (PFAS) exhibit high persistence in the environment and accumulate within the human body, warranting a thorough assessment of their toxicity. In this study, we exposed mice (male C57BL/6J mice aged 8 weeks) to a composite of nine PFAS, encompassing both long-chain PFAS (e.g., perfluorooctanoic acid and perfluorooctanesulfonic acid) and short-chain PFAS (e.g., perfluorobutanoic acid and perfluorobutanesulfonic acid). The exposure concentrations of PFAS were equivalent to the estimated daily human intake in the composition reported (1 µg/L (sum of the nine compounds), representing the maximum reported exposure concentration). Histological examination revealed hepatocyte vacuolization and irregular hepatocyte cord arrangement, indicating that exposure to low levels of the PFAS mixture causes morphological changes in liver tissues. Transcriptome analysis revealed that PFAS exposure mainly altered a group of genes related to metabolism and chemical carcinogenesis. Machine learning analysis of the liver metabolome showed a typical concentration-independent alteration upon PFAS exposure, with the annotation of substances such as glutathione and 5-aminovaleric acid. This study demonstrates that daily exposure to PFAS leads to morphological changes in liver tissues and alters the expression of metabolism- and cancer-related genes as well as phospholipid metabolism.

## 1. Introduction

Per- and poly-fluoroalkyl substances (PFAS) include thousands of chemical compounds containing the perfluoroalkyl moiety C_n_F_2n+1_– [1]. Globally, more than 1,000,000 tons of PFAS, including hydrofluorocarbons, are produced annually [2]. Their unique chemical properties, such as heat and chemical resistance, water and oil repellency, emulsifying characteristics, and light absorption, make them suitable for a broad range of applications. However, some PFAS exhibit exceptional persistence in the environment and readily bioaccumulate in the human body, raising concerns about their health implications and earning them the moniker “Forever Chemicals” [3]. Consequently, PFAS contamination has garnered global attention, with McDonald’s Corporation pledging to eliminate the use of PFAS in food packaging within the next five years, starting in 2020, and Amazon Corporation announcing a ban on the use of PFAS in food containers in December 2020 [4].

Among the PFAS, perfluorooctanoic acid (PFOA) and perfluorooctanesulfonic acid (PFOS) are of significant concern owing to their high bioaccumulation potential. The geometric mean human serum elimination half-life of PFOS is 4.8 years (95% confidence interval [CI], 4.0–5.8), and that of PFOA is 3.5 years (95% CI, 3.0–4.1) [5]. Hence, the production and use of these substances are regulated internationally. In 2009, PFOS was included in Annex B of the Stockholm Convention on Persistent Organic Pollutants, resulting in general restrictions regarding its production, use, import, and export. In 2019, PFOA and its associated compounds were listed in Annex A, and perfluorohexane sulfonic acid (PFHxS), a related chemical, was listed in Annex A in 2022 [6]. Safety thresholds have been identified for major PFAS. However, the regulatory levels of PFOA and PFOS vary widely among countries and organizations. The European Food Safety Authority (EFSA) has set the derived no-effect level (DNEL) for PFOA and PFOS at 1.5 µg/kg/day and 0.15 µg/kg/day, respectively [7]. These limits have become more stringent in recent years, with EFSA introducing a new total weekly tolerable intake of 4.4 ng/kg BW/week for PFOA, PFOS, perfluorononanoic acid (PFNA), and PFHxS in 2020 [8]. Moreover, the EPA set the lifetime health advisory level for PFOA and PFOS at 70 ng/L in 2016. The lifetime health advisory level represents the concentration of a specific contaminant in drinking water at which no adverse health effects are expected over a person’s lifetime of exposure. However, it was decreased to 0.024 ng/L in June 2022 [9]. In Japan, the Ministry of Health, Labour and Welfare (MHLW) listed PFOS and PFOA as water quality management targets in April 2020 and set a preliminary target of 50 ng/L for the combined presence of both compounds [10]. However, this is a provisional target, and a definite evaluation of the suitability of these limits—based on sufficient scientific evidence—remains pending.

Although the complete mechanism of toxicity of PFAS has not been comprehensively understood, its detrimental effects are characterized by hepatotoxicity, lipid metabolism disruption, hypothyroidism, immunosuppression, reproductive toxicity, and carcinogenicity [11]. Reportedly, one of the mechanisms of PFAS toxicity involves the activation of nuclear receptors, such as peroxisome proliferator-activated receptor (PPAR) α, PPARγ, PPARδ, constitutive androstane receptor, and pregnane X receptor (PXR) [12]. The activation of these receptors contributes to disease pathogenesis [13]. These nuclear receptors are active in the liver, which is the primary target for toxicity. In mice, a dose-dependent increase in liver weight, hepatocyte hypertrophy with vacuole formation, and increased (or loss of) peroxisome proliferation were observed at high body burdens of long-chain PFAS [14]. Studies using cultured cells have indicated a link between PFAS and cancer; for example, PFOA, PFOS, and PFHxS induce cell proliferation and malignant transformation in human mammary epithelial cells [15,16]. Although short-chain PFAS, such as perfluorobutanoic acid (PFBA) and perfluorobutanesulfonic acid (PFBS), have been used as alternatives to long-chain PFAS, studies investigating the effects of PFBS exposure in drinking water in mice have revealed that they alter the liver and intestinal metabolome [17,18]. However, most of these studies were based on exposure to relatively high concentrations of single compounds.

The long half-lives of PFAS in the environment and in vivo suggest that people are exposed to several PFAS at low concentrations over prolonged periods. However, limited studies have simulated the real-world exposure conditions in animal models. One study investigated the effects of a PFAS mixture, which mimics an environment contaminated with PFAS found in ski wax, and found that it altered the dopamine levels in mice [19]. Another study reported that exposure to 2 ng/g PFOS results in stunted embryos of bovine cumulus oocytes [20]. However, the toxic effects of PFAS at the concentrations that humans ingest daily remain unknown. In the real world, it is likely that mixed exposure occurs owing to the fact that multiple PFAS are found in the environment. As the sources of exposure are diverse, including drinking water, food, and dust, estimating the exact amount of exposure is challenging. Pérez et al. assessed the PFAS concentrations in food in different regions of the world and estimated the daily human intake of PFAS based on the region [21]. They reported that the concentration varied across regions, ranging from 30 to 100 ng/kg/d, with the children around the Mediterranean Sea ingesting the highest concentration of PFAS at 100 ng/kg/day.

In the present study, we aimed to investigate the toxic effects of low-concentration mixtures of PFAS to which humans can be exposed. We set 100 ng/kg/day as the maximum anticipated daily intake in humans and evaluated the toxic effects of nine linear PFAS, including long-chain PFAS and short-chain PFAS, in mice orally for 5 weeks (Table 1). We hypothesized that toxic effects could occur under exposure to mixed contaminants, even if the concentration of individual components in the mixture is high or if it includes short-chain PFAS. 

## 2. Materials and Methods

### 2.1. Materials

Nine major linear-chain PFAS were tested in this study: PFOA, PFBA, PFOS, PFPeA, PFNA, PFHxA, PFBS, PFHpA, and PFHxS. Their exposure concentrations and compositions were in accordance with a previous study [21]. Table 1 presents the compositions of the different compounds of PFAS. PFOA, PFOS, and PFHxS were purchased from Sigma-Aldrich (St. Louis, MO, USA), whereas the other chemicals were obtained from Tokyo Chemical Industry Co., Ltd. (Tokyo, Japan).

### 2.2. Animal Experiment

Seven-week-old male C57BL/6J mice (N = 15) were purchased from CLEA Japan Inc. (Tokyo, Japan). They were housed under a 12/12 h light/dark cycle at 20–23 °C. Food (CE-2; CLEA) and water gel (HydroGel, ClearH_2_O, Westbrook, ME, USA) were provided ad libitum, and the mice were not subjected to fasting either before or during the experiments. To reduce the risk of an unexpected droplet spread of PFAS-containing drinking water, HydroGel was used as the vehicle/hydration source for the exposure test instead of pure water. HydroGel is 98% water and is subject to rigorous quality control by the company, including assessment of other constituents such as mineral content, ensuring reliability. Health observations were performed thrice per week, and the cages, food, and HydroGel were changed once per week. After one week of adaptation, the mice were randomly divided into three groups, with five individuals in each group.

One group was treated with clean HydroGel (the control group), whereas the other two were treated with HydroGel containing nine linear PFAS at total concentrations of 1 µg/L and 50 µg/L, respectively (PFAS-Low and PFAS-High groups, respectively). The average body weight of mice at the beginning of the experiment was 30 g, and the average daily drinking volume was 3 mL. Hence, the average daily intake of each mouse in the PFAS-Low and PFAS-High groups was estimated at approximately 100 ng/kg/day and 5000 ng/kg/day, respectively. After the 5-week exposure period, mice were euthanized through the inhalation of an excess amount of isoflurane and laparotomized. The organs were harvested from the posterior vena cava after complete blood sampling. All animal care and experimental procedures were approved by the Animal Care Committee of Kitasato University School of Veterinary Medicine (Approval No. 21-012) and were conducted in accordance with the committee and national regulations.

### 2.3. Tissue Histology

Four organs, including liver, spleen, kidney, and heart, were obtained from each mouse and immersed and fixed in a 10% neutral formalin phosphate-buffered solution for at least 1 month, then embedded in Pathoprep568 (Fujifilm Wako Pure Chemical Corporation, Tokyo, Japan) following routine methods. The paraffin blocks were cut into thin sections (4 µm) and stained with hematoxylin and eosin for morphological observation or with periodic acid–Schiff (PAS) with or without amylase digestion for glycogen detection in liver sections, according to the method of Kovac et al. [22].

### 2.4. Liver Transcriptome Analysis

A small piece of liver immersed in RNAlater™ solution (Sigma-Aldrich) was used for RNA extraction. RNA was extracted using TRI reagent (Cosmo Bio Co., Ltd., Tokyo, Japan) and the Monarch Total RNA Miniprep Kit (New England BioLabs Inc., Ipswich, MA, USA) according to the manufacturer’s instructions. The RNA concentration in the extract was quantified by measuring the absorbance at 260 nm using a BioSpectrometer kinetic instrument (Eppendorf, Hamburg, Germany). Subsequent RNA sequence analysis (RNA-seq) of the purified RNA was performed by GenScript Biotech Corp. (Piscataway, NJ, USA). Briefly, RNA was randomly fragmented and reverse transcribed into cDNA, and adapter sequences were attached to both ends of the fragments. The fragments were amplified using polymerase chain reaction (PCR), and fragments with sizes of 200–400 base pairs were selected and sequenced using a NovaSeq 6000 system (Illumina Inc., San Diego, CA, USA). Changes in gene expression were analyzed using the Kyoto Encyclopedia of Genes and Genomes (KEGG) pathway [23].

### 2.5. Real-Time Quantitative PCR

The gene expression levels of eight genes representing pathways that varied in the transcriptome analysis were confirmed via quantitative PCR (qPCR), according to our previous report [24]. Briefly, reverse transcription from mRNA was performed using the LunaScript^®^ RT SuperMix Kit (New England BioLabs), following standard protocols. cDNA was amplified using Luna^®^ Universal qPCR Master Mix and corresponding primers for RT-qPCR. These primers were designed using the Primer Design Tool of the National Center for Biotechnology Information. The primer sequences are shown in Table S1. The PCR conditions included the following: initial denaturation, 95 °C for 60 s; 40 cycles of denaturation, 95 °C for 15 s; and annealing and extension, 60 °C for 30 s. Real-time PCR was performed for each sample using the StepOnePlus™ Real-Time PCR System (Thermo Fisher Scientific). β-actin (*Actb*) was used as the internal control, and the fold change was calculated using the 2^−∆∆Ct^ method.

### 2.6. Metabolome Analysis of Liver

The metabolome was extracted by adding 400 μL of methanol containing internal standards (25 μM N,N-diethyl-2-phenylacetamide and d-camphor-10-sulfonic acid) and 400 μL of ultrapure water to 100 mg of liver sample. Thereafter, the mixture was homogenized using BioMasher^®^ II equipped with PowerMasher^®^ II (Nippi, Incorporated, Tokyo, Japan). The homogenates were centrifuged at 14,000× rpm for 5 min after adding 100 μL of methanol containing internal standards (100 μM of N,N-diethyl-2-phenylacetamide and d-camphor-10-sulfonic acid). After centrifuging, the supernatant was transferred to Amicon^®^ Ultra-0.5 3 kDa filter columns (Merck Millipore, Burlington, MA, USA) and centrifuged at 14,000× rpm for 1 h. The filtrates were transferred to glass vials for ultra-performance liquid chromatography-quantitative time-of-flight mass spectrometry (UPLC/QTOF-MS) analysis. The analysis was performed on an ExionLC AD UPLC system interfaced with an X500R LC-QToFMS system (SCIEX) using the Sequential Windowed Acquisition of All Theoretical Fragment Ion Mass Spectra (SWATH) method. In SWATH, a duty cycle included a single MS scan (accumulation time, 200 ms) followed by a series of product ion scans (accumulation time, 40 ms each) of 30 isolation Q1 windows of modified mass units from m/z 50 to 800. MS/MS spectra were acquired at 35 eV with a collision energy spread of 15 eV. The positive and negative mode injection voltages were set to 5500 and −4500 V, respectively. All samples were analyzed using UPLC with the Agilent InfinityLab Poroshell 120 HILIC-Z (2.7 µm, 50 × 2.1 mm) (Agilent Technologies, Santa Clara, CA, USA) in both positive and negative ion modes for metabolome analysis. The mobile phase for positive mode measurement consists of A: water with 0.1% formic acid, and B: acetonitrile with 0.1% formic acid. For the negative mode, the measurement consists of A: water with 10 mM ammonium bicarbonate, pH adjusted to 9 using 28% ammonia, and B: acetonitrile. For both ion modes of metabolome analysis, the gradient program was as follows: 95% solvent B, then at t = 6 min: 40% B, t = 9 min: 5% B, t = 9.01–11 min: 90% B, and t = 11.01–18 min: 95% B. A serum quality control (QC) sample for metabolome analysis was prepared by pooling and mixing equal volumes of serum samples. QC and blank samples (ultrapure water + internal standard) were injected at intervals of 6–7 sample injections to identify sample carryover and to check for stability during the entire analytical sequence.

In this study, if the peak intensity in the sample was less than three times that of the blank samples, the peak in the sample was considered not detected, and peaks with a detection rate of less than 50% in the actual sample were removed from the data analysis. The coefficient of variation values of below 30% of the metabolites among the analyzed results of QC samples and annotation level 2 (probable structure: matched by high-resolution MS and MS/MS library spectrum) proposed by Schymanski et al. [25] were used for data analysis. Peak heights were normalized to the peak heights of the internal standards, locally weighted least-square regression (locally estimated smoothing function), and cubic spline with QC samples. The metabolome data were analyzed using Mass Spectrometry–Data-Independent Analysis (MS-DIAL) software version 4.90 [26] and R statistical environment v.4.2. Mass spectra were searched against the RIKEN library, MS-bank North America, the NIST20 tandem mass spectrometry library, and the human metabolome database [27]. Candidates with total scores based on the isotope ratio and accurate mass MS/MS similarity were annotated, and an annotation was made for the candidate with the highest score. The annotation cutoff score was set at 80. If the annotated metabolome was apparently an artificial chemical or plant-derived component, the one with the second-best annotation score was adopted.

### 2.7. Molecular Docking

One of the possible mechanisms of PFAS toxicity is binding to the nuclear receptors PPARα, PPARγ, and PPARδ [11,12]. Molecular docking simulations were performed to evaluate the binding affinity between the nine PFAS and three PPARs. The 3D molecular structures of the small molecules and known PPAR ligands were obtained from PubChem. PubChem CIDs are listed in Table 1. They were further preprocessed by adding partial atomic charges to their structures, as determined by molecular mechanics minimization calculated using the Gasteiger method [28]. The 3D structures of murine PPARα, PPARδ, and PPARγ were constructed using the protein 3D structure modeling algorithm Alphafold2 [29]. The protein structure was preprocessed by adding hydrogen atoms and energy minimization using the CHARMM force field [30]. The binding pocket of each PPAR was determined using the coordinates of the pocket with the largest volume identified with the grid-based HECOMi finder [31]. Molecular docking was performed using AutoDock Vina software to determine the best docking poses and docking scores [32]. The search area was a 25 Å × 25 Å × 25 Å grid box, and each calculation was performed 5000 times. The 3D structures obtained were converted to 2D structures using LIGPLOT+ v.2.2.5 to compare the respective amino acid binding modes [33].

### 2.8. Biomarkers of Liver Injury

Liver injury and lipid metabolism biomarkers were measured in plasma samples collected after euthanasia at the end of PFAS exposure. Alkaline phosphatase (ALP) and cholesterol levels were measured colorimetrically using LabAssay™ (FUJIFILM Wako Pure Chemical Corporation, Osaka, Japan), according to standard protocols. Aspartate aminotransferase (AST) levels were measured colorimetrically using a method established by Sigma-Aldrich (MAK055).

### 2.9. LCMS Measurement of PFAS

The concentration of PFAS in the HydroGel was measured using UPLC/QTOF-MS (Table 1). The PFAS mixture (1 µg/L, total concentration) was diluted to 10 ng/L with methanol containing 4 ppb 13CPFOA as an internal standard. The mobile phase conditions for LC were as described by Reza et al. [34], and the details are as follows: Acclaim RSLC C18 column (2.1 × 100 mm, 2.2 μm, Thermo Fisher Scientific) was used for sample separation. The mobile phase is (A) H_2_O:MeOH (90:10) with 5 mM ammonium acetate and (B) MeOH with 5 mM ammonium acetate. The gradient program was started with 1% (0 min, 0.2 mL/min)–1% (1 min, 0.2 mL/min)–39% (3 min, 0.2 mL/min)–99.9% (14 min, 0.4 mL/min)–99.9% (16 min, 0.48 mL/min)–1% (16.1 min, 0.2 mL/min)–1% (21.1 min, 0.2 mL/min). The injection volume was adjusted to 5 μL. Analysis was performed on an ExionLC AD UPLC system interfaced with an X500R LC-QToFMS system (SCIEX) using the Sequential Windowed Acquisition of All Theoretical Fragment Ion Mass Spectra (SWATH) method. In SWATH, a duty cycle included a single MS scan (accumulation time, 400 ms) followed by a series of product ion scans (accumulation time, 90 ms each) of 15 isolation Q1 windows of modified mass units from m/z 25 to 1000. MS/MS spectra were acquired at 35 eV with a collision energy spread of 15 eV. The negative mode injection voltage was set to −4500 V. PFAS was quantified using a 6-point calibration curve in the range of 0–5 ppb. For PFPeA and sulfonates, their precursor ion peaks were used for quantification since their product ion peaks were less sensitive.

### 2.10. Data Analysis

Statistical analyses were performed using JMP Pro 16 software (SAS Institute, Cary, NC, USA). Tukey’s HSD test was used to compare measurements between all groups; a *p*-value of <0.05 was considered statistically significant. For the omics analysis, the false discovery rate (FDR) was adopted to adjust *p*-values, and the results were analyzed using different packages in R v.4.2. A quantitative evaluation of histological specimens ensued employing Python 3.9, amalgamating PyImageJ and OpenCV. Positive domains within Periodic Acid–Schiff (PAS)-stained micrographs underwent meticulous scrutiny through the delineation of luminance-abundant regions within inverted depictions, subsequently quantifying the extracted area. The derivation of RGB values emanated from 50 randomly ascertained coordinates situated within the confines of the cytoplasm. Leveraging these RGB values as salient attributes, a Support Vector Machine (SVM) endowed with multivariate analytical proficiency was invoked to effectuate binary categorization between the cohort subject to the PFAS exposure and the comparator control assembly. The instantiation of model parameters entailed the establishment of C = 1 and γ = 100, with subsequent 5-fold cross-validation undertaken to ascertain the model’s resilience and its capacity for generalized applicability. MetaboAnalyst R was used for the enrichment analysis of the metabolome [35]. The data were normalized by dividing the mean of all samples within each compound by the standard deviation. Enrichment analysis was performed based on the KEGG pathway. Random forest, a machine learning classification method, was utilized to characterize the metabolomics after PFAS exposure [36]. PFAS-Low and PFAS-High groups were merged as PFAS-exposed groups, and a binary classification model was constructed to investigate the differences between the control and PFAS-exposed groups based on metabolomics. The implementation was based on the scikit-learn decision tree library with 5-fold cross-validation. n_estimator was set to 1000, and max_depth was set to 3.

## 3. Results

### 3.1. Histological Examination

**Figure 1:**
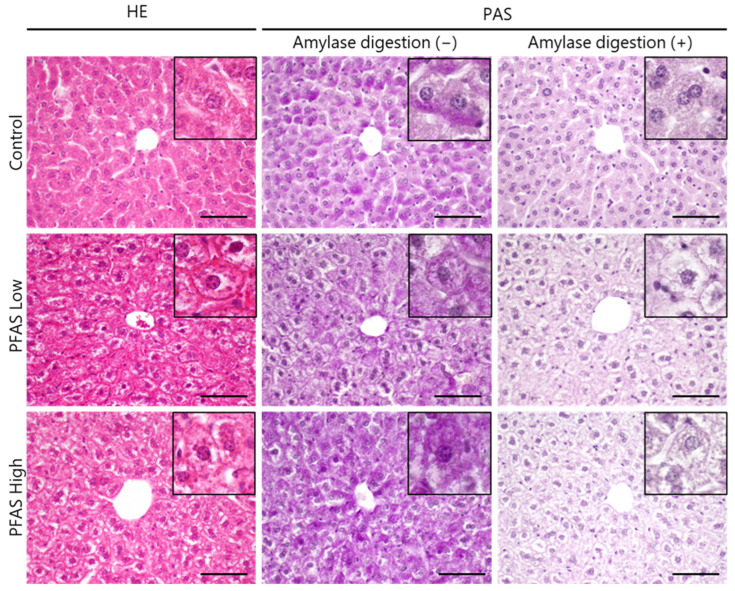
Histology of the liver determined via hematoxylin and eosin (HE) staining of samples in the control, per- and polyfluoroalkyl substances (PFAS)-Low (100 ng/kg/day), and PFAS-High (5000 ng/kg/day) groups. Diffuse vacuolation and hypertrophy of hepatocytes with eosinophilic granules were observed in all mice in the PFAS-Low and -High groups (N = 5, each). Representative images of livers stained with periodic acid–Schiff (PAS) stain with/without amylase digestion. Scale bars = 100 μm.

The most remarkable changes were observed in the liver, with irregular alignment of the hepatic plates in all PFAS-exposed individuals. The cytoplasm tended to have granular vacuolation and appeared pale (Figure 1). No other symptoms, such as inflammation or apoptosis, were observed in any samples. Glycogen degeneration causes similar granular degeneration. Further, the results of the PAS reaction with amylase digestion showed that before the amylase digestion test, the control group was diffusely positive for the PAS reaction and that the PFAS-Low group had a decreased degree of reaction; however, there were no significant differences in the area values of PAS stain-positive regions between groups (Figure S1A). After the amylase digestion test, all groups were completely negative for the PAS reaction (Figure 1). The color tone of the cytoplasmic region was quantified in the PAS-stained image after amylase digestion, when the cell boundaries are most clearly defined (Figure S1B). The PFAS-exposed group was classified as having a lighter color tone in the cytoplasm and was classified as having a different color tone from the control group with 93.3% accuracy when classified via SVM. No distinct histological changes were observed in the spleen, kidney, or heart (Figure S2). There were no significant changes in body weight, clinical symptoms, or gross organ abnormalities prior to euthanasia.

### 3.2. Liver Transcriptome Analysis

**Figure 2:**
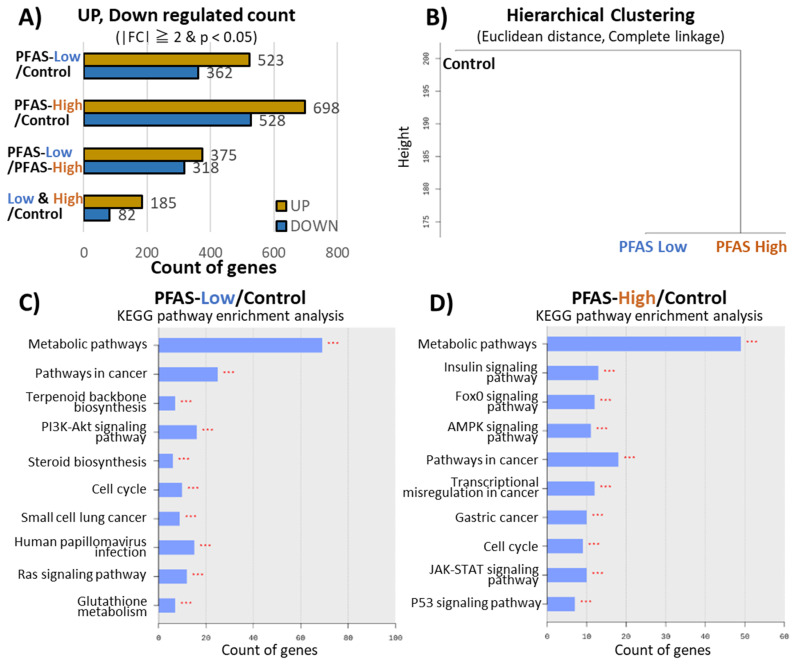
Transcriptome analysis of the liver. (**A**) Shows the number of upregulated and downregulated genes based on the comparison pair’s fold change (FC). (**B**) The high-expression similarities were grouped together using each sample’s normalized value. (Distance metric = Euclidean distance; Linkage method = Complete Linkage.) (**C**) Kyoto Encyclopedia of Genes and Genomes (KEGG) enrichment analysis between the PFAS-Low and control groups. (**D**) KEGG enrichment analysis between the PFAS-High and control groups. ***; *p* < 0.001 detected by false discovery rate-adjusted *t*-tests.

Subsequent experiments were focused on the liver, in which significant histological changes were observed following PFAS exposure. Figure 2A shows the number of genes with more than two-fold changes in expression between each PFAS-Low and PFAS-High groups as detected via RNA-seq. The number of genes that were significantly changed from the control group by two-fold or more in common between PFAS-Low and PFAS-High were 185 (increased) and 82 (decreased) genes. The top 10 most significantly altered genes are listed in Tables S2 and S3. The clustering of these variations with Euclidean distance showed a similar pattern of gene variation in the PFAS exposure groups compared to the control group (Figure 2B). The fluctuating gene groups were classified by function based on KEGG pathways, and enrichment analysis was performed to show the number of genes fluctuated significantly (Figure 2C,D). Compared to the control group, 70 metabolism-related and 30 cancer-related genes were significantly altered in the PFAS-Low group; approximately 50 metabolism-related and 20 cancer-related genes were significantly altered in the PFAS-High group, with metabolism-related genes accounting for the highest number of alterations in the PFAS-Low and PFAS-High groups, followed by cancer-related genes. The pathways that were commonly ranked high in the two PFAS treatment groups in the enrichment analysis were the metabolic pathways, pathways in cancer, and cell cycles. The two pathways also showed carcinogenesis-related pathways, such as small cell lung cancer (appearing in PFAS-Low), gastric cancer, transcriptional misregulation in cancer, and the p53 signaling pathway (appearing in PFAS-High), respectively.

### 3.3. qPCR

**Figure 3:**
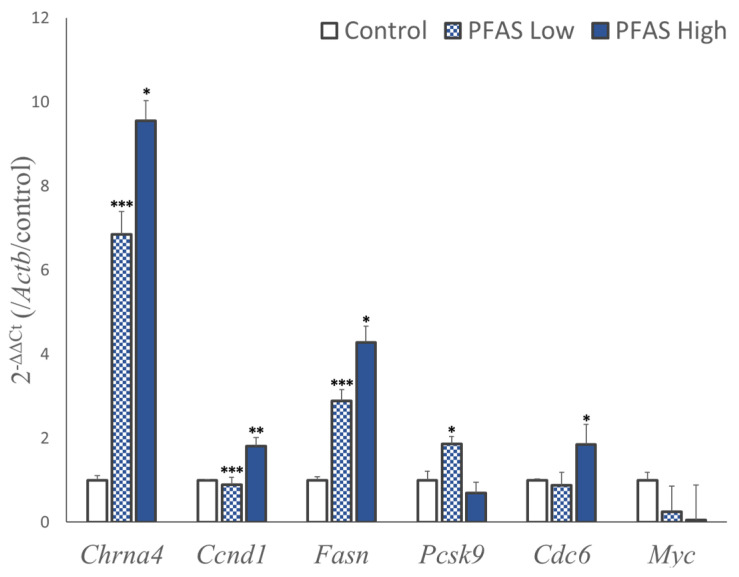
Real-time quantitative polymerase chain reaction. Representative genes of pathways with high variability in RNA sequencing were quantified (N = 5, each group). The y-axis shows the gene expression value relative to the control using β-actin (*Actb*) as the calibration gene. The data are represented as means ± SEM (n = 5). All data were measured in four replicates for each mouse. Asterisks (*) represent significant differences compared with control using Tukey–Kramer’s HSD test—*; *p* < 0.05, **; *p* < 0.01, and ***; *p* < 0.001.

Based on enrichment analysis of the transcriptome, chemical carcinogenesis, cholesterol metabolism, and fatty acid metabolism were selected as pathways with several genes whose expression levels were significantly different in the liver due to PFAS exposure. Six representative target genes of each pathway (cholinergic receptor nicotinic alpha 4 subunit; *Chrna4*, cyclin D1; *Ccnd1*, proprotein convertase subtilisin kexin 9; *Pcsk9*, fatty acid synthase; *Fasn*, cell division cycle 6; *Cdc6*, and c-myc; *Myc*) were quantified and validated in all the individuals (Figure 3). Compared with the control group, the most upregulated gene was *Chrna4*, with a 6-fold and 10-fold increase in expression in the PFAS-Low and PFAS-High groups, respectively (Tukey’s HSD test). Significant increases were observed in *Ccnd1*, a cell cycle regulator that contributes to tumorigenesis, and *Fasn*, a fatty acid synthase that promotes obesity and tumorigenesis, in PFAS-exposed mice. *Pcsk9*, an aggravating factor for blood cholesterol levels, was upregulated only in the PFAS-low group. Conversely, *Cdc6*, an oncogenic gene that regulates DNA replication, was significantly upregulated in the PFAS-High group. The expression levels of *Myc* (c-Myc), an oncogenic gene, did not differ significantly between the control and PFAS-exposed groups.

### 3.4. Metabolomics

**Figure 4:**
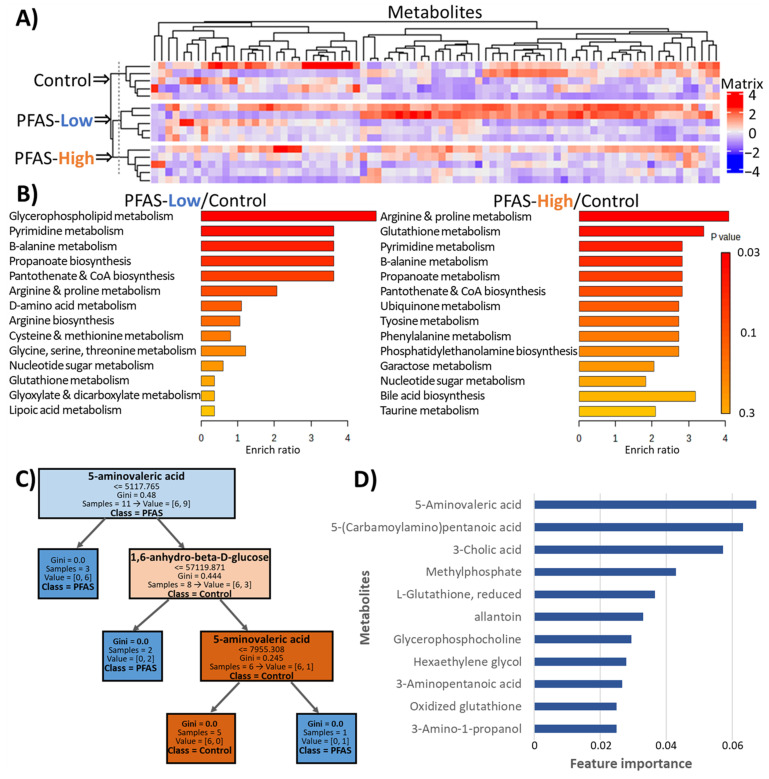
Metabolomics of the liver. (**A**) Heatmap of all 189 metabolites detected in the liquid chromatography–mass spectrometry analysis and hierarchical clustering. (**B**) Quantitative enrichment analysis compared with PFAS-High or PFAS-Low and control groups (N = 5, each). (**C**) An example of a decision tree in a random forest classifier. (**D**) Top 10 features of importance for the random forest classifier (11 compounds were tied for 10th place).

A total of 189 compounds were detected and quantified using metabolomic analysis. Figure 4A shows the heat map of all samples and cluster analysis. The control and PFAS-exposed groups were found to bifurcate at the beginning, and the metabolite variation due to PFAS exposure was similar in the PFAS-Low and PFAS-High groups. Enrichment analysis was performed for 122 of the 189 detected compounds listed in MetaboAnalyst R. Comparison of the PFAS-High and control groups revealed that the ether lipid pathway was the most significantly altered, followed by the glycerophospholipid pathway. A comparison of the PFAS-Low and control groups showed different results, with the nucleotide glucose metabolism pathway being the most significantly altered, followed by the glutathione metabolism pathway (Figure 4B). The glycerophospholipid pathway is a synthetic pathway for the major components of cell membranes, whereas the ether lipid pathway involves a group of lipids derived from the glycerophospholipid pathway that has more physiological activity. The nucleotide sugar pathway involves glycosyl and phosphoglycosyl donors for the biosynthesis of carbohydrates and glycoconjugates in living organisms. However, none of the compounds showed significant differences when their concentrations were compared between the control group and PFAS-exposed groups (false discovery rate [FDR]-adjusted *p* > 0.05).

Hence, to characterize the effects of PFAS exposure on the liver metabolome, a binary classification of PFAS-exposed and control groups based on the metabolome was performed using random forests. The random forest classifier, consisting of the decision tree shown in Figure 4C, discriminated between the PFAS-exposed and control groups from the metabolome with 100% accuracy for the training set and 90% accuracy for the test set. The importance of each feature (compound) in the classifier was calculated. Of the 189 compounds, 114 had a feature importance of zero, indicating that they were not used in the classifier. The 10 most important features are shown in Figure 4D. The results annotated 5-aminovaleric acid as the most important compound. The abundance profiles of the top 10 substances are shown in Figure S3. Among the top 10 compounds, 6 compounds (5-aminovaleric acid, 3-cholic acid, methyl phosphate, L-glutathione, allantoin, and glycerophosphocholine) are registered in the KEGG pathway.

### 3.5. Molecular Docking

**Figure 5:**
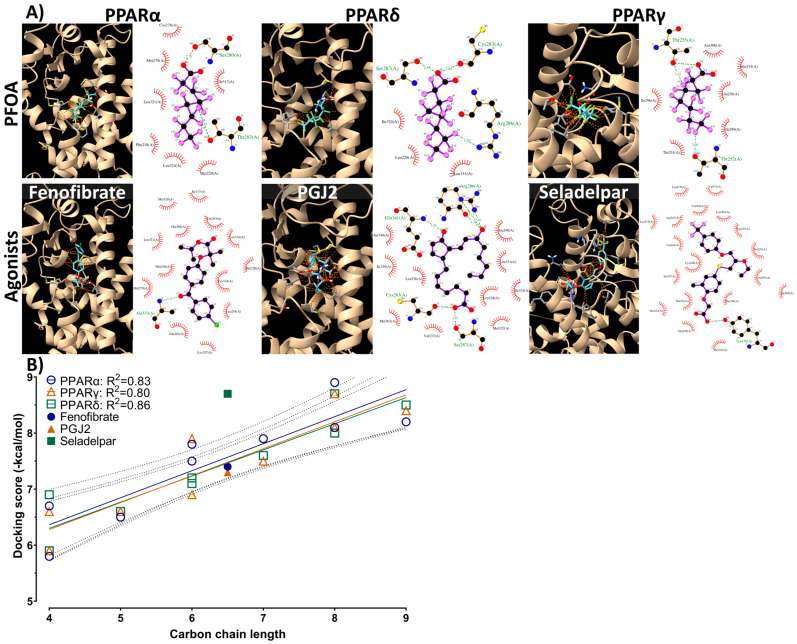
(**A**) Theoretical binding pose obtained via molecular docking simulation for peroxisome proliferator-activated receptor (PPAR) with perfluorooctanoic acid or their known ligands. The protein is shown in ribbon representation with the binding residues shown in stick representation, and orange dashed lines in the 3D diagram indicate ligand–protein interactions. These interactions are visualized as 2D figures using LigPlot+. (**B**) Regression of docking scores and carbon chain length of PFAS. The known ligands are indicated by filled shapes. Linear regression was performed using GraphPad Prism 9. The dashed lines represent the 95% confidence interval of the regression shown as colored lines. The docking scores are shown in Table S4.

The binding energy of each PFAS to the peroxisome proliferator-activated receptor (PPAR), the receptor responsible for chemically responsive hepatocyte hypertrophy, was evaluated using molecular docking. Known ligands for each PPAR were also docked for comparison [37,38,39] (Figure 5, Table S4). PFOS (C8) exhibited the highest binding energy for all PPARα, PPARγ, and PPARδ, followed by PFNA (C9) and PFOA (C8). Compounds with carbon chain lengths above PFHxA (C6) showed higher docking scores with known ligands and docking scores equal to or higher than those of known ligands for PPARs. The linear correlation between these PFAS carbon chain lengths and docking scores showed a positive correlation with high linearity and R^2^ values of ≥0.80 (Figure 5B). Comparing the binding positions of PFOA and the known ligands of PPARs, PFOA was docked into the same binding pocket as the known ligands in all three PPARs (Figure 5B). The number of amino acids in PPARs interacting with the ligands was lower for PFOA than for the known ligands. However, the interacting amino acid species were similar. The binding positions of the eight compounds other than PFOA are shown in Figure S4.

### 3.6. Biomarkers for Liver Injury

**Figure 6:**
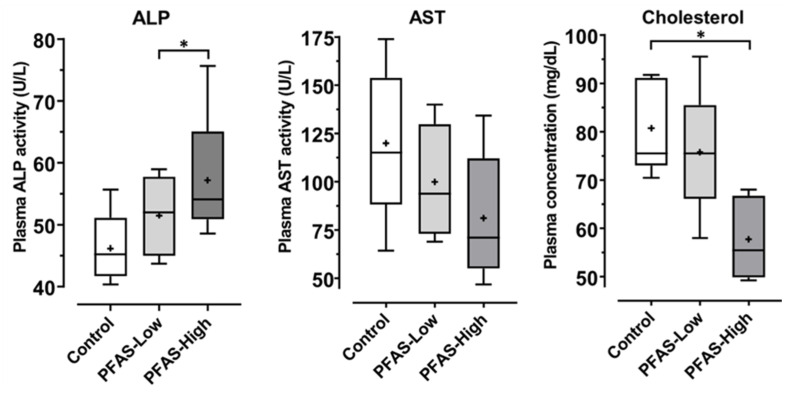
Plasma concentration of hepatic biomarkers, including ALP (alkaline phosphatase), AST (aspartate aminotransferase), and cholesterol in plasma (N = 5, each group). These concentrations were measured using colorimetric methods. The data are graphically represented as box-and-whisker plots, where the boxes represent each quartile along the median, + indicates the mean value, and the whiskers extend to the maximum and minimum values. All data points were measured in duplicate. * denotes significant differences detected using Tukey–Kramer’s HSD test (*p* < 0.05).

Although the levels of ALP, AST (common liver biomarkers), and cholesterol (a lipid metabolism marker) were within the reference range in all three groups [40,41], ALP levels were significantly higher in the PFAS-High group than in the PFAS-Low group (Figure 6). In contrast, cholesterol levels were significantly lower in the PFAS-High group than in the control group.

## 4. Discussion

PFAS have become ubiquitous in the human population, raising concerns regarding their impact on human health. The concentrations of PFOA and PFOS in the human plasma range from 1 to 30 ng/mL [42]. It is imperative to evaluate the effects of real-world, low-concentration mixed exposures. In the present study, mice were orally exposed to nine linear PFAS, including PFOA and PFOS, over a five-week period. Histological examinations revealed significant alterations in the liver tissues in both the PFAS-Low and PFAS-High groups (Figure 1 and Figure S2). The alterations were characterized by vacuolar degeneration featuring eosinophilic granules and an anomalous arrangement of hepatic sinusoids. Hepatocyte vacuolation was frequently observed in the hepatic lobule, particularly near the hepatic portal vein, suggesting that PFAS is taken up via the gastrointestinal tract and directly affects hepatocytes proximal to the portal vein. Considering the absence of glycogen denaturation, as indicated by the negative results of PAS staining (Figure 1), we inferred that chemically responsive hepatic hypertrophy was a gross abnormality. Chen et al. (2022) postulated that inflammation and apoptosis of hepatocytes occurred in response to PFOS exposure via drinking water at a concentration of 500 µg/L, with Elcombe et al. (2012) positing that periportal hepatocellular vacuolation in rats could result from PFOS intake of 20 or 100 ppm [18,43]. However, this study demonstrates that even much lower mixed-exposure doses can cause analogous histological alterations.

Omics analysis was performed to evaluate molecular biological alterations in the liver. Transcriptome analysis revealed similar patterns of genetic variation between the PFAS-Low and PFAS-High groups (Figure 2B). The genes were categorized according to their function based on the KEGG pathway. Genes related to metabolism and carcinogenesis were predominantly modified by PFAS exposure (Figure 2C,D). Quantitative PCR was performed to validate the changes in expression levels within these pathways. The transcript with the highest mRNA expression included *Chrna4*, which was upregulated by approximately 10-fold and 6-fold in the high-exposure group and low-exposure group, respectively, compared to the untreated control group (Figure 3). *Chrna4* is located upstream of *Ccnd1*, an oncogene in the KEGG chemical carcinogenesis pathway, suggesting that *Chrna4* contributes to chemical carcinogenesis. In addition, *Fasn*, which is regulated by PPARs, was also significantly upregulated by PFAS exposure. Overexpression of *Fasn* and enhanced lipid metabolism promote cancer cell growth and metastasis because cancer cells utilize fatty acids for tumor growth and metastasis [44]. Hence, PFAS exposure enhanced lipid metabolism and promoted tumorigenesis. Moreover, *Ccnd1* and *Cdc6* genes—which regulate cell proliferation during the cell cycle—also exhibited increased expressions in the exposure groups. *Ccnd1* triggers the G1–S phase transition in the cell cycle by activating cyclin-dependent kinases (*Cdk4* and *Cdk6*) and enhances the proliferation of cancer cells [45]. *Cdc6*, a cell growth regulator, is expressed in the quiescent phase (G1 phase) in normal cells but is expressed at all cell cycle stages in cancer cells and enhances cancer cell proliferation. Our results suggest that even low concentrations of the PFAS mixture increase the expression of these genes. The carcinogenic risk of low-concentration PFAS exposure needs to be evaluated in longer-term chronic exposure studies. The majority of genes with the highest expression variation in RNA sequencing were lnc-RNAs and did not significantly impact the enrichment analysis (Tables S2 and S3). However, when these genes were analyzed with Enrichr [46], the results indicated the involvement of the Regulation of Gene Expression By Hypoxia-inducible Factor R-HSA-1234158 in Reactome 2022 and the Proteins Involved In Gene Expression By Hypoxia-inducible Factor R-HSA-1234158 in the Elsevier pathway. These findings suggest that PFAS exposure may induce hypoxia-like changes in gene expression that were not characterized by KEGG-based analysis.

Metabolome analysis revealed similar patterns of metabolites altered with PFAS in the PFAS-Low and PFAS-High groups (Figure 4A). However, enrichment analysis suggested that different pathways were altered in these groups, with the glycerophospholipid and ether lipid pathways particularly affected in the PFAS-Low group. The main factor contributing to this alteration in the PFAS-Low group includes the approximately 0.6-fold decrease in glycerophosphocholine (α-GPC) levels (Figure S5A). Hence, the initial decrease in acetylcholine precursor α-GPC levels after PFAS exposure may have been compensated by an increase in the expression of the acetylcholine receptor, *Chrna4* (Figure 3). The glutathione metabolic pathway, a defense mechanism against oxidative stress, was upregulated in the PFAS-High group. The nucleotide sugar pathway was upregulated in both PFAS-exposed groups owing to an approximately two-fold increase in glucose 6-phosphate levels (Figure S5B). G6P is the starting substrate of the pentose phosphate pathway, an NADPH-producing pathway in the liver. This increase may have occurred in response to NADPH requirements for glutathione. In contrast, an increase in the reduced and oxidized glutathione levels (Figure S3) suggested that the oxidative stress response was not disrupted and that the glutathione levels increased adaptively. It should be noted that not all individual variations for each compound were statistically significant. While the enrichment analysis showed different results among the exposure groups, binary classification via machine learning, in addition to cluster analysis, discriminated PFAS exposure from the metabolome with 90% accuracy, suggesting exposure-specific effects. The substance that contributed the most to the classification, 5-aminovaleric acid, is known to act as a weak GABA receptor agonist [47,48]. GABA release is regulated by nicotinic acetylcholine receptors, and elevated liver concentrations of 5-aminovaleric acid from PFAS exposure may be associated with increased gene expression of *Chrna4*. On the other hand, 5-aminovaleric acid had the second-highest concordance in the metabolome annotation of this study, and the compound with the best concordance was 2,5-dihydro-2,4-dimethyloxazole, a component of nuts (Figure S6). 5-(Carbamoylamino)pentanoic acid, which had the second highest contribution in the random forest model, was also initially annotated with the pharmaceutical piracetam. Information on 5-(carbamoylamino)pentanoic acid is limited, but it is a derivative of citrulline (2-amino-5-(carbamoylamino)pentanoic acid), which is involved in the urea cycle, having undergone deamination. As a limitation of this study, it should be noted that the compounds were not precisely identified using analytical standards but were annotated based on their MSMS spectra. However, since the random forest classification did not use the physiological function of the compounds, they were still uniquely observed in the PFAS-exposed groups. More detailed mass spectrometric identification of these compounds is warranted. The decision tree machine learning method used to classify the exposure groups effectively evaluated the toxicity effects in a *p*-value-independent manner based on compounds with unknown biological functions. In contrast, the glycerophospholipid pathway revealed via RNA-Seq analysis did not show any significant changes in gene expression. These findings suggest that multi-omics analysis may be advantageous for detecting pre-pathological changes.

Hepatic precancerous proliferative alterations are influenced by various etiological factors, with the activation of nuclear receptors, PPARs, being one of the primary factors. In mammals, three types of PPARs have been identified: PPARα, PPARγ, and PPARδ. These receptors participate in cell differentiation, energy metabolism, and cancer cell growth [49]. In our study, we conducted docking simulations to evaluate the binding affinity between nine PFAS and mouse PPARα, PPARγ, and PPARδ. The results revealed a robust correlation between the length of the PFAS carbon chain and binding affinity for all three PPAR isotypes in mice (Figure 5B). Notably, PFOS, PFNA, and PFOA exhibited binding poses similar to those of fenofibrate, a known PPARα ligand, and exhibited even higher binding potency than that of fenofibrate (Table S4). This suggests that PFAS can act as PPARα agonists. Interestingly, even short-chain PFAS demonstrated bioactivity, as docking scores equal to or above −7.0 generally indicate high pharmacological activity in nanomolar orders, which is a significant factor in drug discovery screening [50]. A previous study reported that PFOA, PFOS, and short-chain PFAS can activate PPARα and PPARγ in vitro [51]. Short-chain PFAS have relatively low toxicity but may contribute to PPAR-dependent hepatic hypertrophy. Although hepatocyte hypertrophy was observed in the present study, the RNA-seq results did not show a substantial increase in the expression of PPAR-responsive genes (e.g., a 2.0-fold increase in *Cyp7a1* expression in the PFAS-High group). However, the increased expression of cell proliferative genes, including *Ccnd1*, in the oncogenic pathway indicates the presence of other receptor-binding-mediated cell proliferation signals. To accurately assess the toxicity of PFAS, it is crucial to elucidate the key AOP events related to hepatocyte hypertrophy.

Plasma ALP, AST, and cholesterol levels were measured as biomarkers of liver injury (Figure 6). Although ALP levels increased significantly following exposure to high concentrations of PFAS, they remained within the normal range. AST and cholesterol levels were within the normal range and decreased with increasing exposure. These findings suggest that PFAS exposure did not cause a severe liver injury, although histological alterations were evident in the liver. Hepatocytes transiently enlarge as an adaptive response to chemical exposure, whether adverse or nonadverse [52]; therefore, histological changes are considered adaptive hypertrophy. As carcinogenic genetic changes were observed in this study, there is a risk of liver damage with prolonged exposure. Chronic toxicity studies of mixed exposures are essential to elucidate this point. Species differences should be considered when extrapolating the findings of the present study to humans. A previous study showed that the sensitivity of PPARα to PFAS is higher in mice than in humans and that PFAS at levels that cause significant carcinogenicity in rodents may be insensitive or unresponsive in humans [53]. However, the half-lives of PFOA, PFOS, and other PFAS are shorter in mice than in humans, and the same exposure may result in higher residue concentrations in humans [11]. Therefore, it is essential to consider more appropriate models for evaluating the health effects of PFAS on humans. Susceptibility to PFAS may also vary by strain of mouse, with the finding that C57BL6 mice are more prone to hypercholesterolemia on PFOA and high-fat diets than BALB/c mice [54]. In the present study, plasma cholesterol levels tended to be rather low in the PFAS-exposed group, but further validation is required. In addition, C57BL/6J and C57BL/6N differ in their response to non-alcoholic steatohepatitis (NASH), even within the same C57BL/6 strain. In the tetrachloromethane-induced NASH model, BL/6J mice exhibited more severe liver fibrosis, whereas in the high-fat diet-induced NASH model, BL/6N mice had more weight gain and liver injury than BL/6J mice [55]. One limitation of this study is that it involved mixed exposure; therefore, it is unknown whether there were individual or synergistic effects of each substance. Assessment of the mixed exposure effects of chemical substances is an international issue, and there is currently no established assessment method [56]. Further validation using both the experimental validation demonstrated in this study and theoretical prediction models is expected.

## 5. Conclusions

The findings of this study highlighted that low concentrations of PFAS mixtures can cause changes in gene expression (including cancer-related genes) and histology even after a relatively short exposure period of 5 weeks. In the future, exposure studies must be conducted over longer periods and at lower concentrations to reflect the environmental exposure conditions.

## Figures and Tables

**Table 1:** Composition of the nine poly-fluoroalkyl substances (PFAS) used in the study.

Compound	Abbreviation	%	Expected Concentration (ng/L)	Measured Concentration (ng/L)	Carbon Chain Length	Pubchem CID
Perfluorooctanoic acid	PFOA	25	250	264	8	9554
Perfluorobutanoic acid	PFBA	20	200	298	4	9777
Perfluorooctanesulfonic acid	PFOS	15	150	113	8	74,483
Perfluorononanoic acid	PFNA	10	100	115	9	67,821
Perfluorovaleric acid	PFPeA	6	60	99	5	75,921
Perfluorohexanoic acid	PFHxA	6	60	72	6	67,542
Perfluorobutanesulfonic acid	PFBS	6	60	99	4	67,815
Perfluoroheptanoic acid	PFHpA	6	60	35	7	67,818
Perfluorohexanesulfonic acid	PFHxS	6	60	35	6	67,734
SUM		100	1000	1130		

The expected concentration indicates the concentration of PFAS in the hydrogel to which the PFAS Low group was exposed, and the measured concentration is the concentration of PFAS in the gel, as measured via mass spectrometry.

## Data Availability

Data are available upon request.

## Supplementary Materials

The following supporting information can be downloaded at https://www.mdpi.com/article/10.3390/toxics12010052/s1, Figure S1. Colorimetric determination of hepatocyte cytoplasm. Figure S2. Histological examination of the kidney, heart, and spleen via hematoxylin and eosin staining. Figure S3. Box-and-whisker plot of the top 10 essential compounds in the random forest classifier. Figure S4. Representative 2D diagram of binding poses between peroxisome proliferator-activated receptors (α, δ, and γ) and poly-fluoroalkyl substances (PFOS, PFHxS, and PFBS) obtained via molecular docking. Figure S5. Box-and-whisker plot of glycerophosphocholine and D-glucose 6-phosphate, which contributed to the enrichment analysis shown in Figure 5B. Figure S6. MSMS spectra of representative metabolite. Table S1. Primer information. Table S2. Top 10 genes upregulated after PFAS exposure detected via RNA-sequence. Table S3. Top 10 genes downregulated after PFAS exposure detected via RNA-sequence. Table S4. Docking scores.
