# Supplementary material for: Toxicity Assessment of Mixed Exposure of Nine Perfluoroalkyl Substances at Concentrations Relevant to Daily Intake
Source: Toxics. 2024 Jan 10;12(1):52. doi: 10.3390/toxics12010052 (PMC10819949; doi:10.3390/toxics12010052)
Supplement: Supplementary file 1 [file toxics-12-00052-s001.zip › toxics-2734568-supplementary.pdf]

toxics

Supplementary Materials: Toxicity Assessment of Mixed Exposure of Nine Perfluoroalkyl Substances at Concentrations Relevant to Daily Intake

Kazuki Takeda, Taki Saito, Sakura Sasaki, Akifumi Eguchi, Makoto Sugiyama, Kio Suzuki, Saeka Eto, and

Ryo Kamata

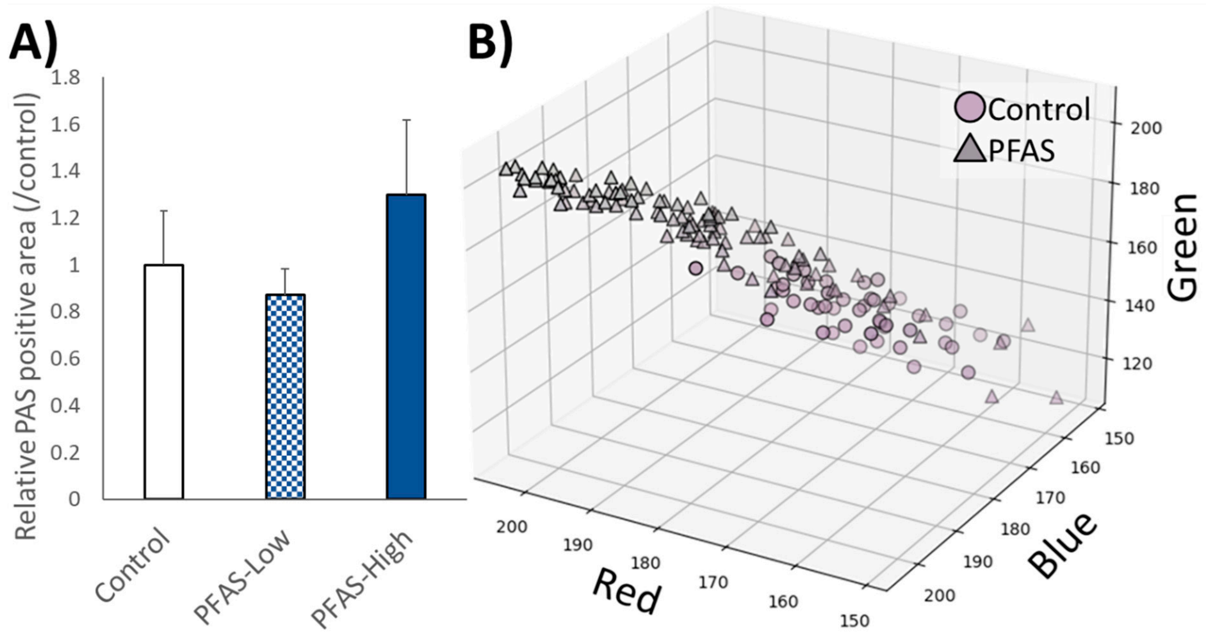

Figure S1. A) Area value of PAS stain positive region. The values are shown relative to the mean value of the control group (mean ± SD, N=5). No significant differences were detected in all substances (Tukey Kramer's HSD test, $p > 0.05$). B) Colorimetric determination of hepatocyte cytoplasm. RGB values of 50 points were mechanically obtained from cytoplasmic regions in the images of periodate Schiff (PAS) stain with amylase digestion (Fig. 1).

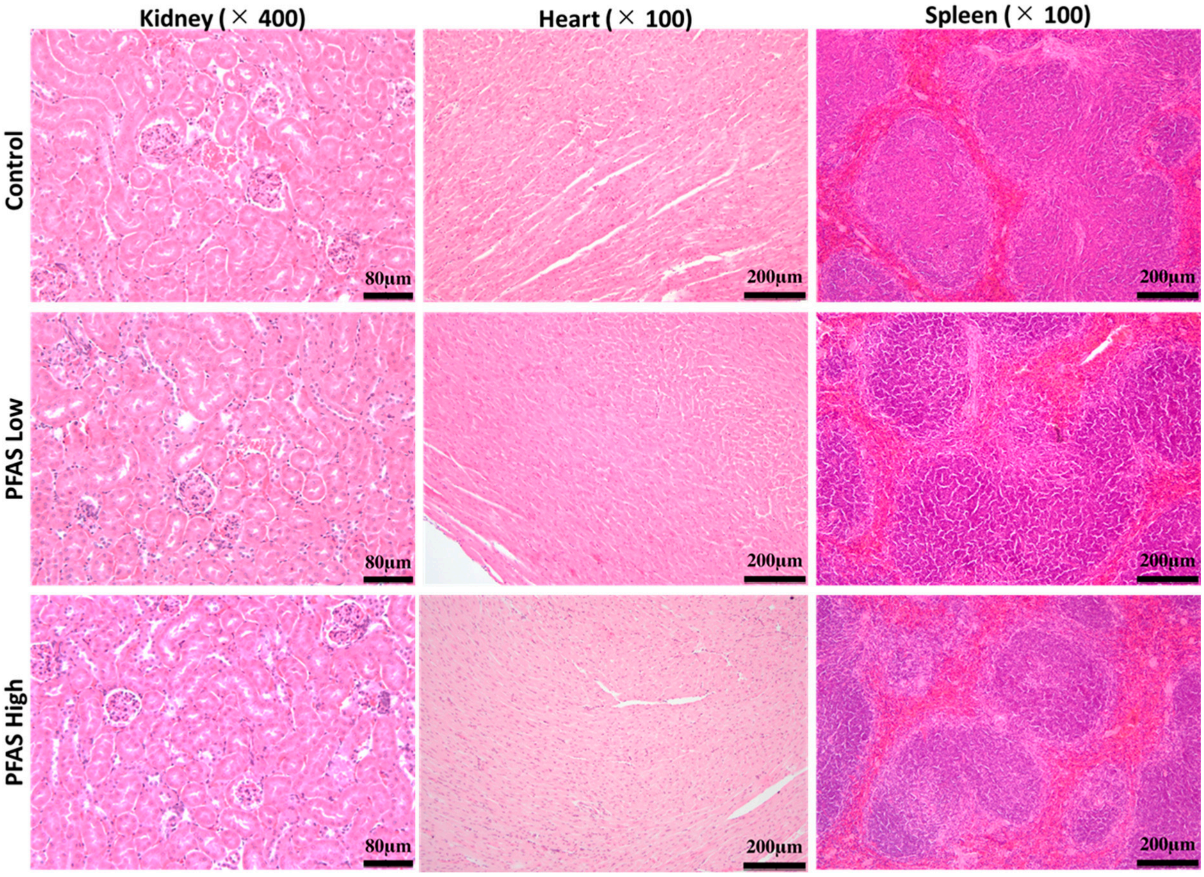

Figure S2. Histological examination of the kidney, heart, and spleen by hematoxylin and eosin (HE) staining.

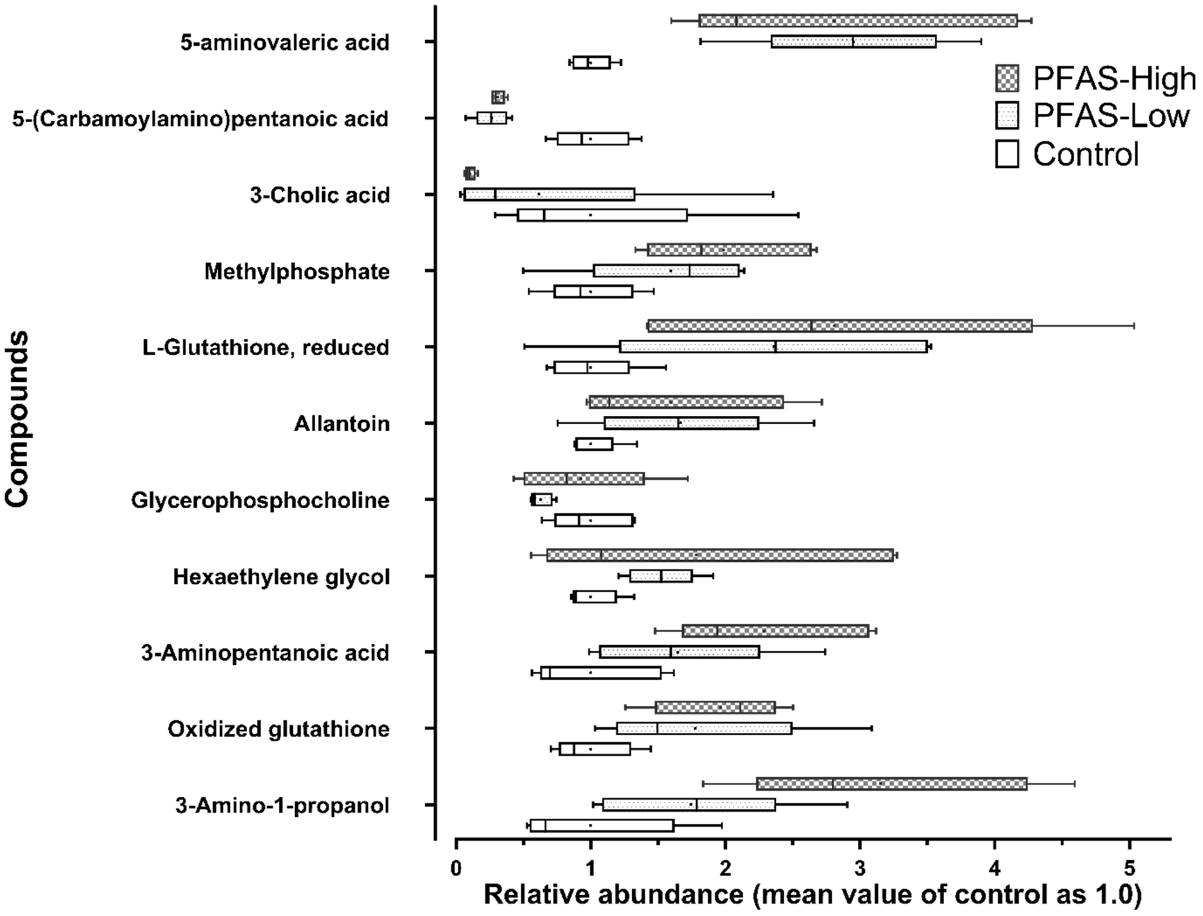

Figure S3. Box-and-whisker plot of the top 10 essential compounds in the random forest classifier shown in Fig. 4D. The x-axis represents the relative abundance corrected by dividing it by the control group's mean for each compound (the mean of the control should be 1.0). + indicates mean values. No significant differences were detected in all substances (FDR adjusted $p > 0.05$).

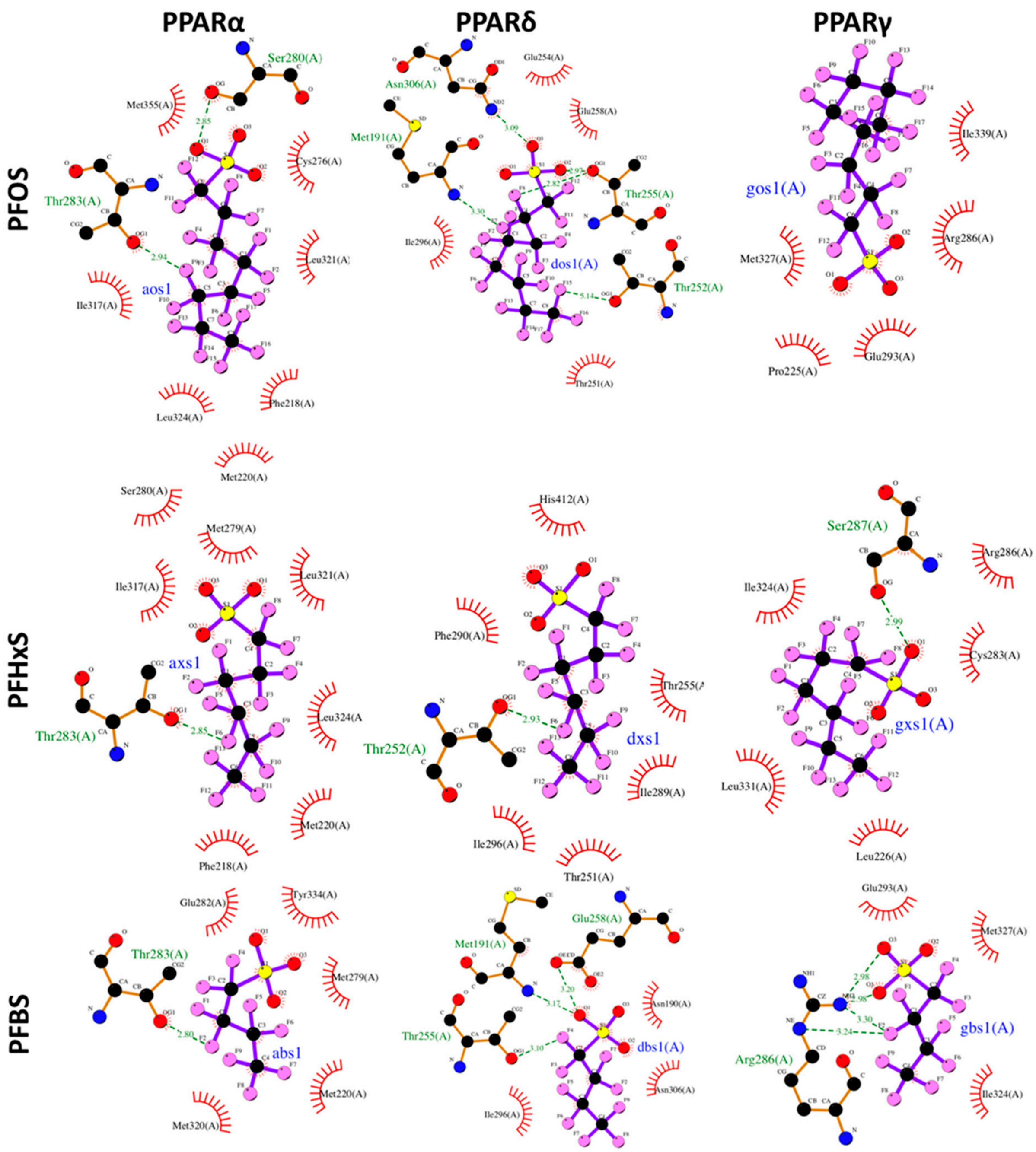

Figure S4. Representative 2D diagram of binding poses between peroxisome proliferator-activated receptors (α, δ, and γ) and poly-fluoroalkyl substances (PFOS; perfluorooctanesulfonic acid, PFHxS; Perfluorohexanesulfonic acid, and PFBS; perfluorobutanesulfonic acid) obtained by molecular docking.

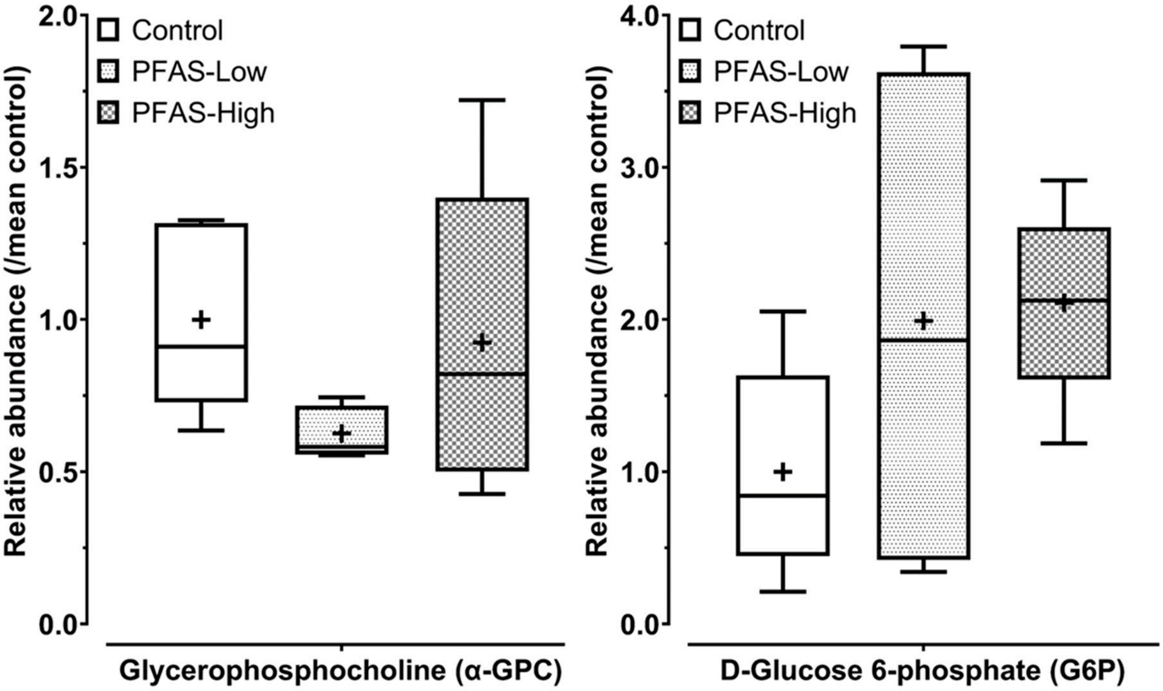

Figure S5. Box-and-whisker plot of glycerophosphocholine (α-GPC) and D-glucose 6-phosphate, which contributed to the enrichment analysis shown in Fig. 4B. The y-axis represents the relative concentration corrected by dividing it by the mean of the control group. + indicates mean values. No significant differences were detected in all substances (false discovery rate adjusted $p > 0.05$).

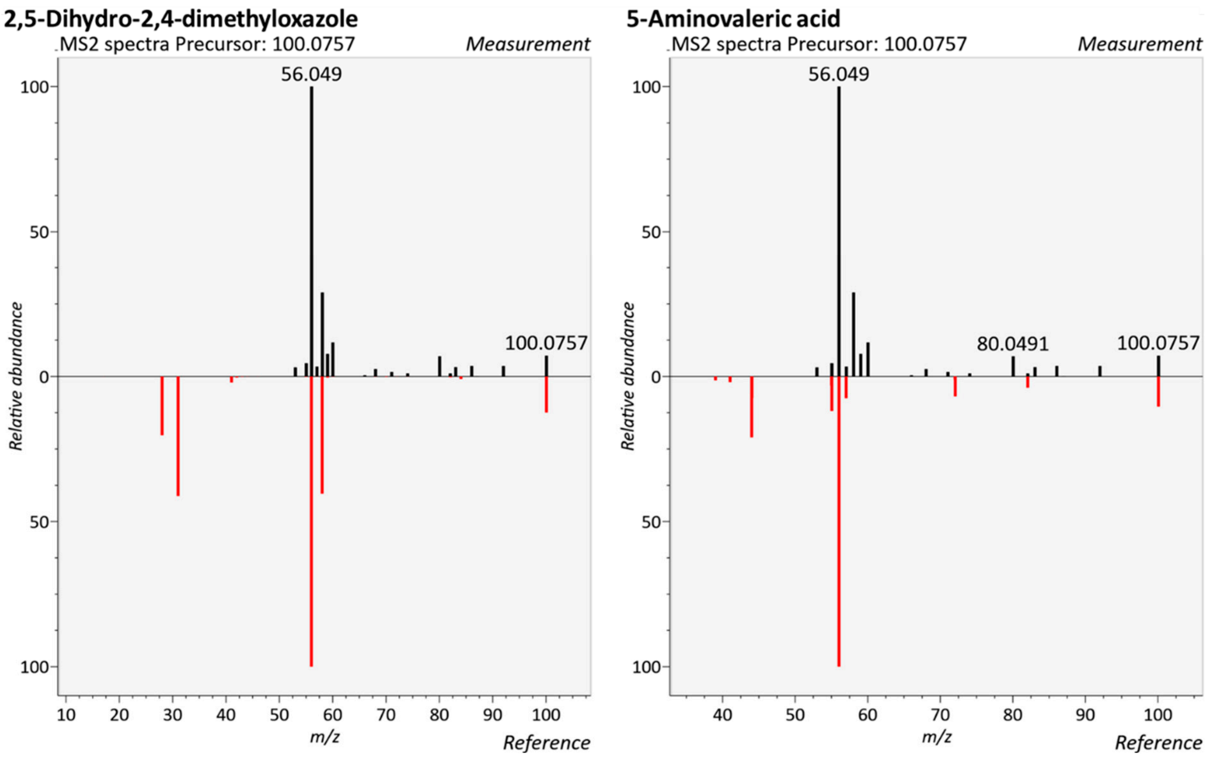

Figure S6. MSMS spectra of representative metabolite. The upper black peaks are the measured value, and the lower red ones are the reference. 2,5-Dihydro-2,4-dimethyloxazole had the highest annotation score based on MSMS similarity (score = 87.25), but was rejected because it is a plant-derived compound. 5-Aminovaleric acid had the second highest annotation score (score = 80.06), is an endogenous metabolite, and was selected as an annotation for this MSMS spectrum.

Table S1. Primer information. All primers were designed using the National Center for Biotechnology Information Primer designing tools. Amplification efficiency was determined by six points of 5-fold step dilution (number of replicates = 4).

Gene (Product size)	Sequences	Amplification efficiency (%) Standard error of mean
Chrna4	Forward：GTGTGGGTGAAGCAGGAGTG	103.4
(177 bp)	Reverse：GGTGGGTGACTGCAAAGTCC	3.2
Ccnd1	Forward：CATTTCCAACCCACCCTCCA	97.9
(152 bp)	Reverse：CCAGGGCCTTGACCGGG	4.5
Pcsk9	Forward：ATCACCGACTTCAACAGCGT	90.2
(174 bp)	Reverse：GCCCTTCCCTTGACAGTTGA	2.3
Fasn	Forward：CCTTCGGTTCAGTCTCTTTCCA	90.6
(111 bp)	Reverse：ACACCCTCCAAGGAGTCTCAC	1.7
Cdc6	Forward：TTACGGTGGTGATCGAGACG	96.9
(103 bp)	Reverse：TGATGGCCACACAACTCTCTG	4.3
Myc	Forward：GTTGGAAACCCCGCAGACAG	99.1
(94 bp)	Reverse：ATAGGGCTGTACGGAGTCGT	3.5
Actb	Forward：ACTGTCGAGTCGCGTCCA	97.9
(86 bp)	Reverse：ATCCATGGCGAACTGGTGG	0.6

Table S2. Top 10 genes upregulated after PFAS exposure detected by RNA-sequence.

Transcript_ID	Gene_Symbol	Gene_biotype	Protein_ID	PFAS-Low/Control Fold change	PFAS-Low/Control Log10 p value	PFAS-High/Control Fold change	PFAS-High/Control Log10 p value
XR_001778663	Gm32006	lncRNA	.	11.69	-27.12	8.46	-20.00
XR_001778560	Gm35254	lncRNA	.	9.73	-19.24	5.86	-10.56
XR_871801	Gm32872	lncRNA	.	9.62	-4.06	7.82	-3.65
XR_379628	Gm33459	lncRNA	.	9.41	-32.79	6.39	-22.85
NM_130448	Pcdh18	protein_coding	NP_569715.3	8.35	-6.07	4.80	-2.74
NM_001001180	Zfp941	protein_coding	NP_001001180.2	8.10	-5.17	4.00	-1.63
XR_003948291	Gm39299	lncRNA	.	7.42	-1.95	5.34	-1.39
XR_385146	Gm34854	lncRNA	.	6.96	-2.37	78.44	-41.41
XR_877087	Gm30284	lncRNA	.	6.41	-7.65	9.73	-14.75
NM_001361910	Serpina9	protein_coding	NP_001348839.1	6.12	-3.24	5.11	-2.64

Top 10 genes out of 185 genes with a >2-fold increase in expression in PFAS-Low and PFAS-High in common compared to the control, with a corrected p-value less than 0.05.

Table S3. Top 10 genes downregulated after PFAS exposure detected by RNA-sequence.

Transcript_ID	Gene_Symbol	Gene_biotype	Protein_ID	PFAS-Low/Control		PFAS-High/Control	
				Fold change	Log10 p value	Fold change	Log10 p value
NR_004843	Gm44502	lncRNA	.	-37.30	-26.29	-42.09	-29.05
NR_033450	Serpina3h	transcribed_pseudogene	.	-15.08	-24.60	-93.32	-38.95
XR_003951569	Gm41349	lncRNA	.	-8.41	-4.06	-4.29	-2.53
NM_033576	Pcdhgb4	protein_coding	NP_291054.1	-7.31	-3.20	-6.63	-3.19
XR_003955335	Gm42270	lncRNA	.	-7.00	-3.98	-3.43	-2.47
NM_023612	Esm1	protein_coding	NP_076101.1	-6.69	-5.48	-4.56	-4.62
NM_001162950	Hif3a	protein_coding	NP_001156422.1	-6.65	-2.70	-4.34	-2.03
NM_001142959	Bcl2l15	protein_coding	NP_001136431.1	-6.43	-2.53	-3.28	-1.40
XR_003955939	LOC115486295	lncRNA	.	-5.99	-2.20	-8.91	-3.09
NM_026840	Pdgfrl	protein_coding	NP_081116.3	-5.85	-4.84	-13.04	-8.84

Top 10 genes out of 82 genes with a >2-fold decrease in expression in PFAS-Low and PFAS-High in common compared to the control, with a corrected *p*-value less than 0.05.

Table S4. Docking scores.

Compound	C length	Docking score (kcal/mol)		
		PPARα	PPARγ	PPARδ
PFOS	C8	8.9	8.7	8.7
PFNA	C9	8.2	8.4	8.5
PFOA	C8	8.1	8.1	8
PFHpA	C7	7.9	7.5	7.6
PFHxS	C6	7.8	7.9	7.2
PFHxA	C6	7.5	6.9	7.1
PFBS	C4	6.7	6.6	6.9
PFPeA	C5	6.5	6.6	6.6
PFBA	C4	5.8	5.9	5.9
Agonists	-	7.4	7.3	8.7

Theoretical docking scores were calculated by molecular docking. The representative docking poses are shown in Figure 5 and S3. Typical agonists of each peroxisome proliferator-activated receptor (PPAR) were docked into the PPARs for comparison. Agonists for PPARα; fenofibrate, PPARγ; prostaglandin J2, PPARδ; seladelpar.
